# Supplementary material for: Association between Dysfunction of the Nucleolar Stress Response and Multidrug Resistance in Pediatric Acute Lymphoblastic Leukemia
Source: Cancers (Basel). 2022 Oct 19;14(20):5127. doi: 10.3390/cancers14205127 (PMC9601175; doi:10.3390/cancers14205127)
Supplement: Supplementary file 1 [file cancers-14-05127-s001.zip › Supplemental Figure S1.pdf]

Supplementary information

Association between dysfunction of the nucleolar stress response and multidrug resistance in pediatric acute lymphoblastic leukemia

Shunsuke Nakagawa [1], Kohichi Kawahara [2,*], Yasuhiro Okamoto [1,*], Yuichi Kodama [1], Takuro Nishikawa [1], Yoshifumi Kawano [1], and Tatsuhiko Furukawa [2]

1. Department of Pediatrics, Graduate School of Medical and Dental Sciences, Kagoshima University, Kagoshima 890-8520, Japan
2. Department of Molecular Oncology, Graduate School of Medical and Dental Sciences, Kagoshima University , Kagoshima 890-8520, Japan

a

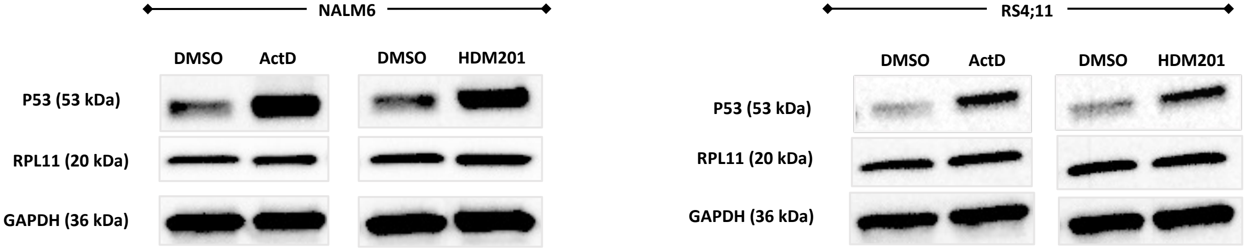

Cell line	NALM6			
Drug	DMSO	ActD	DMSO	HDM201
P53/GAPDH	0.226	1.087	0.200	0.520
RPL11/GAPDH	0.597	0.730	0.665	0.840

Cell line	RS4;11			
Drug	DMSO	ActD	DMSO	HDM201
P53/GAPDH	0.119	0.237	0.139	0.548
RPL11/GAPDH	1.703	1.472	1.265	0.906

b

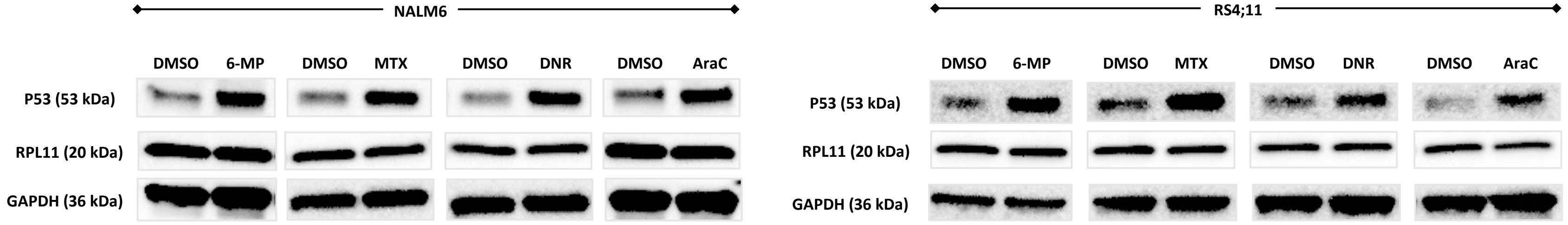

Cell line	NALM6							
Drug	DMSO	6MP	DMSO	MTX	DMSO	DNR	DMSO	AraC
P53/GAPDH	0.095	0.163	1.079	1.771	0.685	1.607	0.096	0.159
RPL11/GAPDH	0.320	0.258	3.606	2.522	2.177	2.401	0.251	0.285

Cell line	RS4;11							
Drug	DMSO	6MP	DMSO	MTX	DMSO	DNR	DMSO	AraC
P53/GAPDH	0.629	1.257	0.429	1.235	0.341	0.400	0.249	0.297
RPL11/GAPDH	5.920	6.076	3.921	3.093	2.685	2.070	2.529	1.527

Figure S1. Actinomycin D, HDM201, 6-mercaptopurine, methotrexate, daunorubicin, and cytarabine increased TP53 protein expression. (a) Western blot analysis of TP53 and RPL11 at 24 h after 1 nM ActD or 100 nM HDM201 treatment in NALM6 cells as well as after 48 h of 0.1 nM ActD or 10 nM HDM201 treatment for RS4;11; (b) Western blot analysis of TP53 and RPL11 after 24 h of drug treatment in NALM6 cells and 48 h of drug treatment in RS4;11 cells. GAPDH levels were used as a loading control. Each cell was treated with 100 μM 6-mercaptopurine, 100 nM methotrexate, 10 nM daunorubicin, and 100 nM cytarabine for NALM6 cells as well as with 10 μM 6-mercaptopurine, 100 nM M methotrexate, 3 nM daunorubicin, and 3 nM cytarabine for RS4;11 cells. 6-MP, 6-mercaptopurine; ActD, actinomycin D; AraC, cytarabine; DMSO, dimethyl sulfoxide; DNR, daunorubicin; MTX, methotrexate.
